# The Emergence of *Klebsiella pneumoniae* with Reduced Susceptibility against Third Generation Cephalosporins and Carbapenems in Lagos Hospitals, Nigeria

**DOI:** 10.3390/antibiotics10020142

**Published:** 2021-02-01

**Authors:** Kabiru O. Akinyemi, Rebecca O. Abegunrin, Bamidele A. Iwalokun, Christopher O. Fakorede, Oliwia Makarewicz, Heinrich Neubauer, Mathias W. Pletz, Gamal Wareth

**Affiliations:** 1 Department of Microbiology, Lagos State University, Km 15, Badagry Expressway, P.O. Box 0001 Lasu Post Office, Ojo, Lagos 102101, Nigeria; rabegunrin@gmail.com (R.O.A.); kriskorede@yahoo.co.uk (C.O.F.); 2 Molecular Biology & Biotechnology Department, Nigerian Institute of Medical Research, Yaba, Lagos 100001, Nigeria; bamwal@yahoo.com; 3 Institute for Infectious Diseases and Infection Control, Jena University Hospital, Am Klinikum 1, 07747 Jena, Germany; Oliwia.Makarewicz@med.uni-jena.de (O.M.); mathias.pletz@med.uni-jena.de (M.W.P.); 4 Friedrich-Loeffler-Institut, Institute of Bacterial Infections and Zoonoses, Naumburger Str. 96a, 07743 Jena, Germany; heinrich.neubauer@fli.de; 5 Faculty of Veterinary Medicine, Benha University, Moshtohor, Toukh 13736, Egypt

**Keywords:** *Klebsiella pneumoniae*, Carbapenem, ESBL, Emergence, Resistance, Nigeria

## Abstract

This study investigated the prevalence of *Klebsiella* (*K*.) *pneumoniae* isolates among clinical samples of patients in four medical centers in Lagos, Nigeria and the burden of extended-spectrum beta-lactamases (ESBL) and carbapenem-resistant *K. pneumoniae* (CRKP) strains. Different samples (stool, blood, urine, wound swabs and nasal swabs) from 127 patients with suspected Gram-negative infections based on on-site performed Gram-stain from four public hospitals between March and September 2015 were analyzed. *K. pneumoniae* was identified in 43 (34%) patients. Resistance rates of these 43 strains according to the CLSI breakpoints were as followed: cotrimoxazole (90.7%), cefuroxime (74.4%), ofloxacin (55.8%), ceftazidime (46.5%), and cefixime (35%). Three isolates (7%) were resistant to imipenem. All isolates were susceptible to amoxicillin/clavulanic acid and nitrofurantoin. The prevalence of ESBL-producing, MDR and CRKP strains was 69.8%, 62.8%, and 7.0%, respectively. Of the ESBL-producing isolates, two *K. pneumoniae* isolates obtained from urine harbored both *bla*_SHV_ and *bla*_CTX-M-1_, and a third isolate from urine harbored only the *bla*_CTX-M-1_. This study revealed the emergence of CRKP isolates and *bla*_CTX-M-1_ and *bla*_SHV_ co-harboring *K. pneumoniae* strains in Lagos hospitals. The emergence of CRKP strains is an early warning signal for carbapenem antibiotics’ prudent use with concern for their efficacies.

## 1. Introduction

*Klebsiella* (*K*.) *pneumoniae* is a Gram-negative opportunistic nosocomial bacterial pathogen. It is involved in several localized and disseminated hospital-acquired infections such as burns infections, sepsis, respiratory and gastrointestinal tract infections, urinary tract infections, pyogenic liver abscesses, and soft tissue and wound infection [1]. The emergence of carbapenem-resistant *K. pneumoniae* (CRKP) strains has become an ultimate challenge for public health globally due to their ability to disseminate rapidly in the hospital environment and their extended antibiotic resistance phenotypes [2]. In 2001, the first *K. pneumoniae* isolate with KPC-2 production was identified in the USA [3]. There are many mechanisms in *K. pneumoniae* that can drive carbapenem resistance; KPC-2 production is just one. A few years later, outbreaks began to appear in several countries. Nowadays, it is the most common carbapenemase-producing *Enterobacterales* (CPE), and is considered one of the most rapidly growing global threats due to the high mortality in hospital-associated infections [4]. In 2017, CRKP was classified among those global critical pathogens listed by WHO concerning discovering and developing new antibiotics. 

In Nigeria, *K. pneumoniae* was among the most common causes of lower respiratory tract infections [5], neonatal septicemia, and bacteremia in children [6,7]. Antimicrobial susceptibility testing of 306 Gram-negative isolates from patients admitted to three teaching hospitals in South Western Nigeria in 2011 and 2013 revealed resistance to third-generation cephalosporins in 92.2% and carbapenems in 52.6% of isolates [8]. Examination of 108 *K. pneumoniae* and 140 *E.coli* isolates from two tertiary hospitals in northwest Nigeria showed that 58% of the isolates were ESBL producers, while resistance to meropenem was observed in 40.3% of the isolate, and resistance to imipenem was observed in 36.6% [9]. However, the prescription and use of carbapenem antibiotics are still low, and there is a lack of recent information on multidrug resistance (MDR)-associated *K. pneumoniae.* The present study was conducted to investigate the prevalence of *K. pneumoniae* among clinical samples of patients in four medical centers in Lagos, Nigeria, and determine the burden of extended-spectrum beta-lactamase- (ESBL) and carbapenemase-producing strains.

## 2. Results and Discussion

*Klebsiella pneumoniae* was isolated from 34% (43/127) of clinical samples analyzed in this study. *K. pneumoniae* was present in about half (55.8%) of urine samples screened, an indication of etiological diagnosis in urinary tract infections, as no other bacterial pathogens were isolated. *K. pneumoniae* was detected in a quarter (23.3%) of nasal swabs. In these cases, the link between *K. pneumoniae* isolation and infection cannot be considered sufficiently strong for an etiologic diagnosis because other Gram-positive bacterial agents could not be ruled. Three strains of *K. pneumoniae* (6.98%) were isolated from three patients with soft tissue infection. Three strains (6.98%) were isolated from three gastroenteritis cases, of which no other bacterial agents were detected (Table 1). *K. pneumoniae* was detected in samples of all the age groups studied, with young adults (15–30) being the age group most at the risk of *K. pneumoniae*-associated infections, occurring in 27.9% (12/43) followed by 25.6% (11/43) of patients in the age group 5–14 years (Table 1). The prevalence of *K. pneumoniae* among patients suffered from urinary tract infections (UTIs) [10], suppurative otitis [11], and gut infection [12] is increasing worldwide. 

In Nigeria, malaria is endemic. Three *K. pneumoniae* strains (6.98%) were isolated from patients with concomitant *Plasmodium falciparum* infection. Co-infections of *Enterobacterales* with *P*. *falciparum* have been reported in some malaria-endemic African countries, including Nigeria, and were associated with increased severity [13]. Thus, such cases were also expected in this study, as it can be suggested that *K. pneumoniae*-associated bacteremia caused exacerbation of asymptomatic or silent malaria. Hence, this is the first report on *K. pneumoniae-Plasmodium* spp. co-infections in patients within Lagos hospitals in recent times.

Most isolates were resistant to cotrimoxazole (90.7%), followed by cefuroxime (74.4%), ofloxacin (55.8%), ceftazidime (46.5%), and cefixime (35%). Three isolates (7%) obtained from urine showed resistance to imipenem with a minimum inhibitory concentration (MIC) > 8 mg/L. Resistance rates were lowest for gentamicin (4.7%), imipenem (7%), followed by ciprofloxacin (28%) and no resistance (0.0%) was recorded for amoxicillin/clavulanic acid and nitrofurantoin (Table 2). *K. pneumoniae* was among the predominant Gram-negative bacteria isolated in Sokoto, Northwest Nigeria, in 2019. It represented 14% of isolated bacteria, and most of the isolates were MDR and exhibited ESBL and carbapenemase activities [14]. Examination of 48 *K. pneumoniae* strains isolated from Medical Institution in Oyo State, Nigeria, showed resistance in 88% of isolates to streptomycin and in 92% to cloxacillin, oxacillin, and colistin [15].

In this study, 27 (62.8%) and 30 (69.8%) *K. pneumoniae* were MDR and ESBL producing strains, respectively (Table 3). Previous epidemiological investigations have shown that MDR *K. pneumoniae* strains harboring ESBL genes exist in environmental sources [16] and are usually associated with nosocomial infections in Nigerian hospitals [17,18]. However, there are still few data available on the prevalence of *K. pneumoniae* in Nigerian hospitals. The published data are limited to regional studies and/or a limited number of samples [19]. Our results are comparable to Olalekan and colleagues’ findings, who identified *K. pneumoniae* in 35.4% of samples collected from four hospitals in Lagos between 2016–2018 [17], and Raji and co-workers, who found that *K. pneumoniae* represented 31.4% of strains collected during an assessment of the prevalence of drug resistance and ESBL among members of the family *Enterobacterales* at Lagos teaching hospital [18].

In these MDR *K. pneumoniae* isolates, eleven resistance patterns were identified. The pattern ceftazidime/cefuroxime/ofloxacin/ciprofloxacin/cotrimoxazole (CAZ-CRX-OFL-CIP-COT) was the most frequent one (Table 4). Two *K. pneumoniae* isolates from urine (U3 and U11) harbored both *bla*_CTX-M-1_ and *bla*_SHV_, and one isolate (U19) harbored only *bla*_CTX-M-1_. None of the tested strains contained the *_bla_*_TEM._ The emergence of resistance to imipenem is a new phenomenon, and the explanation is inconclusive because this drug is rarely used and relatively expensive. The prevalence of carbapenem resistance among clinical isolates of *Enterobacterales* was between 2.8% and 53.6% in a tertiary hospital in Lagos, Nigeria [20]. In 2019, most of *K. pneumoniae* strains isolated in Sokoto, Northwest Nigeria, exhibited ESBL and carbapenemase activities [14]. None of the three ESBL genes were detected in any of the three carbapenem-resistant *K*. *pneumoniae* isolates in our study. However, this may not ultimately rule out that these strains are non-carbapenemase producers, as they might be harboring other carbapenemase gene markers not screened in this study.

Only three genes were checked by PCR due to limited resources, which is a limitation of this study. Interestingly, the three CRKP strains were found in two different hospitals at a close distance of 20 km, an indication of the emergence and circulation of CRKP in Lagos, Nigeria. The prevalence of carbapenem-resistance in 177 isolates of *Enterobacterales* was 22% in South-West Nigeria in 2018; of them, 35.9% (*n* = 14) were *K. pneumoniae* [21]. The emergence of ESBL due to *bla*_CTX_ and *bla*_SHV_ genes in *K. pneumoniae* has also been reported in other recent African studies. A high distribution of ESBL with the dominant *bla*_CTX-M-1_ gene marker was reported in Accra and Kumasi, Ghana [22]. A high prevalence of ESBL-producing *K. pneumoniae* in clinical isolates was reported in Côte d’Ivoire [23]. Strains harboring *bla*_CTX-M-15_ and *bla*_SHV-134_ were isolated from pig and abattoir workers in Cameroon [24]. Carbapenem resistance is emerging in Africa, despite its not being routinely used due to high cost [25]. New antibiotics and new strategies are required to mitigate this increasing threat.

## 3. Materials and Methods

### 3.1. Study Population, Case Definition, Sample Collection and Bacteriology

The prevalence and burden of ESBL and CRKP strains were investigated, involving 127 patients with various types of infections admitted to four public Lagos hospitals between March and September 2015. The Central Public Health Laboratory (CPHL), Lagos State University Teaching Hospital (LSUTH), National Institute of Medical Research (NIMR), and Iba Primary Health Centre (IPHC) contributed 42, 33, 32, and 20 samples, respectively. The institutional review boards of Lagos State University and the Nigerian Institute of Medical Research approved the study and the ethical approval with a code [Ref. No. LREC 06/10/1071] was obtained. Moreover, the consent of the patients was sought. Data of the patients, including age and sex, were noted. In total, 37 urine samples, 30 nasal swabs, and 20 wound swabs were aseptically collected from patients diagnosed with urinary tract infections, otitis media and pneumonia, and soft tissue abscesses. Additionally, 20 fresh feces samples were collected from patients with gastroenteritis, and 4 mL blood was collected from another 20 patients suffering from pyrexia of unknown origin (PUO). All blood samples from PUO patients were subjected to the thick-blood smear technique to detect the malaria parasite (*Plasmodium* spp.). Bacterial culture of all samples was done on brain heart infusion (BHI) and Macconkey agar (Oxoid, UK), and plates were incubated at 37 °C overnight under aerobic conditions. The lactose fermenting discrete colonies were subjected to biochemical identification, according to Crown and Steel 1993 [26]. Colonies biochemically confirmed as *Klebsiella* spp. were subjected to the MICROBACT 24E identification system (Oxoid, UK).

### 3.2. Antimicrobial Susceptibility Testing (AST) 

All confirmed *K. pneumoniae* strains were tested against ten antibiotics by both disc diffusion and microdilution methods according to the CLSI guidelines [27]. The following antibiotic discs were used: cotrimoxazole (COT) (25 μg), ciprofloxacin (CIP) (5 μg), ofloxacin (OFL) (5 μg), gentamicin (GEN) (10 μg), cefixime (CXM) (5 μg), amoxicillin/clavulanic acid (AMC) (30 μg), cefuroxime (CRX) (30 μg), ceftazidime (CAZ) (30 µg), imipenem (IMP) (30 μg), and nitrofurantoin (NIT) (300 μg) (Oxoid, UK). The inhibition zones’ diameters were measured with a ruler and compared with a zone-interpretation chart. The microdilution test was performed with the same set of antibiotics for consistency (Sigma, Deisenhofen, Germany) following the manufacturer‘s instructions. In this study, resistance was defined for isolates exhibiting intermediate resistance and resistance. The multidrug-resistant (MDR) phenotype was defined as acquired non-susceptibility to at least one agent in three or more antimicrobial categories [28].

### 3.3. Screening of Extended-Spectrum Beta-Lactamase (ESBL)

All isolates that exhibited reduced susceptibility and resistance to third-generation cephalosporin were screened for ESBL production using the double disc synergy test (DDST). This was done by placing the 3GC antibiotics, i.e., ceftazidime (30 µg) and ceftriaxone (30 µg), at a distance of 15 mm (center to center) from 30 µg amoxicillin/clavulanic acid (20 µg amoxicillin and 10 µg clavulanic acid) using CLSI interpretation guidelines [27]. Detection of carbapenemase enzyme activity was done following the modified Hodge test (MHT) as described by Landman and colleagues [29]. For three ESBL gene makers, the *bla*_CTX-M-1_ group, *bla*_SHV_, and *bla*_TEM_, PCR-based screening was conducted [30,31]. Briefly, genomic DNA was extracted by the Tris-EDTA boiling extraction method [32], and the concentration and purity of DNA were determined spectrophotometrically (BIO-RAD Smart Spec 3000; Hercules, CA, USA). The detection of β-lactamase-encoding genes carried out using multiplex polymerase chain reaction (PCR) with primers that correspond to conserved regions of *bla*_TEM_, *bla*_SHV_, and *bla*_CTX-M-type_ genes. The primers were synthesized and supplied by Promega Corp, Germany. Each PCR reaction was done in a 20 µL volume, comprising 1 × PCR buffer (pH 8.3), 1.5 mM of MgCl_2_, 200 nM each of the deoxynucleotide triphosphates (dNTPs), 40 picomoles each of the forward primer and reverse primers indicated in Table 5. One microliter (1 µL) each of the genomic DNA templates (~100 ng) and 1.25 U of Taq polymerase (Promega, Germany) were used. The PCR program consisted of an initial denaturation step at 94 °C for 3 min, followed by 25 cycles of DNA denaturation at 94 °C for 30 s, primer annealing at 54 °C for 30 s, and primer extension at 72 °C for 1 min. After the last cycle, a final extension step at 72 °C for 7 min was applied. Five-microliter aliquots of PCR products were analyzed by gel electrophoresis with 2% agarose (Sigma-Aldrich, USA). Gels were stained with ethidium bromide at 0.5 µg/mL and visualized by UV transilluminator. A 100 bp DNA ladder (Fermentas, Burlington, ON, Canada) was used as a marker to extrapolate the 190 bp PCR product. The PCR reaction was done using a TC-312 thermal cycler (Techne, Amsterdam, The Netherlands).

## 4. Conclusions

In conclusion, this study revealed the emergence and dissemination of carbapenem-resistant *K. pneumoniae* isolates and *bla*_CTX-M-1_ group and *bla*_SHV_ co-harboring *K. pneumoniae* strains in Lagos. This study is limited by using a PCR screening tool that could only detect *bla*_CTX-M-1_, *bla*_SHV_ and *bla*_TEM_. CRKP is emerging in Nigeria, despite carbapenem compounds not being routinely used in the health care system due to their high cost. New antibiotics and new strategies are needed to mitigate this increasing threat. A comprehensive study on the real situation on carbapenemase-producing *K. pneumoniae* in Lagos using advanced molecular typing tools to assess the diversity and clonal relatedness of the strains is required to better monitor and understand the spread of MDR *K. pneumoniae* in this region.

## Figures and Tables

**Table 1:** Number and types of collected samples and *K. pneumoniae* positive isolates distributed across the subjects’ age and sex used in the current study sampling hospitals.

Parameters		Types and Number of Collected Samples (Positive Sample)					
		Urine	Nasal Swab	Wound Swab	Feces	Blood	Total
Hospitals	CPHL	9 (6)	11 (4)	7 (1)	9 (1)	6 (1)	42 (13)
	LASUTH	10 (5)	9 (3)	5 (1)	2 (0)	7 (2)	33 (11)
	NIMR	12 (8)	7 (2)	3 (1)	6 (2)	4 (0)	32 (13)
	IPHC	6 (5)	3 (1)	5 (0)	3 (0)	3 (0)	20 (6)
	Total	37 (24)	30 (10)	20 (3)	20 (3)	20 (3)	127 (43)
Age in Year	0–4	6 (3)	3 (1)	2 (0)	3 (0)	1 (0)	15 (4)
	5–14	11 (7)	7 (2)	5 (1)	2 (0)	6 (1)	31 (11)
	15–30	9 (5)	6 (1)	7 (2)	7 (2)	5 (2)	34 (12)
	31–49	5(4)	8 (3)	4 (0)	5 (1)	5 (0)	27 (8)
	≥ 50	6(5)	6 (3)	2 (0)	3 (0)	3 (0)	20 (8)
	Total	37 (24)	30 (10)	20 (3)	20 (3)	20 (3)	127 (43)
Sex	Male	21 (11)	13 (7)	9 (1)	12 (2)	11 (2)	66 (23)
	Female	16 (13)	17 (3)	11 (2)	8 (1)	9 (1)	61 (20)
	Total	37 (24)	30 (10)	20 (3)	20 (3)	20 (3)	127 (43)

The Central Public Health Laboratory (CPHL), Lagos State University Teaching Hospital (LSUTH), National Institute of Medical Research (NIMR), and Iba Primary Health Centre (IPHC).

**Table 2:** Antimicrobial susceptibility data and MIC values of 43 *K. pneumoniae* strains collected from hospitals in Lagos.

Antimicrobial Agent	Standard Range		Test Results			
	S ≤ (mg/L)	R ≥ (mg/L)	S (%)	R (%)	MIC50 (mg/L)	MIC90 (mg/L)
Ciprofloxacin	0.25	0.5	72.1	27.9	0.25	1.5
Ofloxacin	0.25	0.5	44.2	55.8	0.25	0.5
Gentamicin	2	4	95.3	4.7	1.5	2.0
Cefixime	1	2	65.1	34.9	1.0	1.5
Ceftazidime	1	2	53.5	46.5	1.0	1.5
Cefuroxime	8	16	25.6	74.4	16	16
Imipenem	2	8	93.0	7.0	1.0	1.5
Cotrimozaxole	2	4	9.3	90.7	4.0	16
Amoxicillin/Clavulanic Acid	8	16	100	0.0	16	16
Nitrofurantoin	8	16	100	0.0	16	16

S: sensitive; R: resistance, MIC: minimum inhibitory concentration.

**Table 3:** Distribution of *K. pneumoniae* ESBL, MDR, and CAB in relation to clinical samples.

Samples	Samples No. (%)	Positive No. (%)	ESBL+ No. (%)	MDR+ No. (%)	CAB+ No. (%)
Urine	37 (29.2)	24 (55.8)	16 (66.7)	17 (70.8)	3 (12.5)
Nasal swab	30 (23.7)	10 (23.3)	7 (70.0)	4 (40.0)	0 (0.00)
Wound swab	20 (15.7)	3 (6.98)	2 (66.7)	1 (33.3)	0 (0.00)
Feces	20 (15.7)	3 (6.98)	2 (66.7)	2 (66.7)	0 (0.00)
Blood	20 (15.7)	3 (6.98)	3 (100)	3 (100)	0 (0.00)
TOTAL	127 (100)	43 (34%)	30 (69.8)	27 (62.8)	3 (6.98)

ESBL: Extended Spectrum ß-lactamase; MDR: multidrug-resistant; CAB: carbapenem; (+): positive resistance.

**Table 4:** Patterns of MDR to *K. pneumoniae* isolates recovered from clinical samples at hospitals in Lagos.

Sample Types	No. of Isolates	Strains ID	Resistance Pattern
Urine and Blood	5	U12, U36, B37, U38, U42	CRX-OFL-COT
Urine	4	U10, U23, U30, U43	CAZ-CRX-OFL-COT
Feces	1	F31	CXM-OFL-CIP-COT
Urine	1	U7	CRX-CXM-GEN-OFL-COT
Urine	2	U3, U11	CAZ-CRX-CXM-OFL-COT
Urine	2	U13, U35	CAZ-CRX-CXM-IPM-COT
Blood	1	B40	CRX-CXM-OFL-CIP-COT
Urine, Wound, and Nasal Swab	3	W17, U19, N26	CAZ-CRX-OFL-CIP-COT-CXM
Urine	1	U18	CAZ-CRX-OFL-CIP-COT-CXM-IPM
Urine and Nasal Swab	2	U39, N4	CAZ-OFL-CIP-COT
Urine, Nasal Swab, Feces, and Blood	5	B33, N9, N16, F32, U41	CAZ-CRX-OFL-CIP-COT
Total	27		

CAZ: ceftazidime (30 μg); CRX: cefuroxine (30 μg); GEN; gentamicin (10 μg); CXM: cefixime (5 μg); OFL: ofloxacin (5 μg); AUG: amoxicillin/clavulanic acid (30 μg); NIT: nitrofurantoin (300 μg); CIP: ciprofloxacin (5 μg); IPM: imipenem (30 μg); COT: cotrimoxazole (25 μg); U (urine), B (blood), F (feces), W (wound swab), N (nasal swab).

**Table 5:** The primer sequences used for amplification of *bla* genes.

Target	Primer	Primer Sequence (5′-3′)	Product Size (bp)	Ref.
*bla*_TEM_	TEM-F	TCCGCTCATGAGACAATAACC	931	[30]
	TEM-R	TTGGTCTGACAGTTACCAATGC		
*bla*_SHV_	SHV-F	TGGTTATGCGTTATATTCGCC	868	[31]
	SHV-R	GGTTAGCGTTGCCAGTGCT		
*bla*_CTX-M_	CTX-F	TCTTCCAGAATAAGGAATCCC	909	[30]
	CTX-R	CCGTTTCCGCTATTACAAAC		

## Data Availability

Not applicable.
